# Ilheus Virus Infection in Human, Bolivia

**DOI:** 10.3201/eid1803.111486

**Published:** 2012-03

**Authors:** Erika A. Venegas, Patricia V. Aguilar, Cristhopher Cruz, Carolina Guevara, Tadeusz J. Kochel, Jorge Vargas, Eric S. Halsey

**Affiliations:** Naval Medical Research Unit 6, Lima, Peru (E.A. Venegas, P.V. Aguilar, C. Cruz, C. Guevara, T.J. Kochel, E.S. Halsey);; New York University, New York, New York, USA (E.A. Venegas);; Centro Nacional de Enfermedades Tropicales, Santa Cruz, Bolivia (J. Vargas)

**Keywords:** Ilheus virus, flavivirus, viruses, phylogenetics, humans, Bolivia, Bolivian Amazon, mosquitoes

**To the Editor:** Ilheus virus (ILHV) was first isolated from mosquitoes of the genera *Ochlerotatus* and *Psorophora* near Ilheus, Bahia, Brazil, in 1944 (*1*). After its discovery, the virus was also isolated from other mosquito species, including the genera *Culex*, *Sabethes*, *Haemagogus*, and *Trichoprosopon*, and from a variety of birds in different countries in Latin America (*2*). Only a few reports describe isolation of this virus from humans in Central and South America with symptoms ranging from subclinical to severe febrile disease (*2**–**6*). In mild cases, patients often reported gastrointestinal or respiratory symptoms lasting ≈1 week. In severe cases, either the central nervous or cardiac system can be affected. However, long-term sequelae or deaths have not been described. No epidemics attributed to ILHV have been reported.

In November 2005, a 15-year-old boy (farmer) sought medical attention in a health clinic in Magdalena, Bolivia, after having fever for 5 days. The patient’s symptoms included malaise, asthenia, conjunctival injection, vesicular rash, facial edema, arthralgia, myalgias, bone pain, abdominal pain, headache, and earache. Signs of cardiac, neurologic, or renal damage were not detected. A blood specimen was obtained during the clinic visit, and a convalescent-phase sample was obtained 24 days after onset of symptoms. At that follow-up visit, the patient reported a full recovery from his symptoms. Both samples were sent to the Naval Medical Research Unit No. 6 in Lima, Peru, for processing as part of a clinic-based study to determine the etiology of febrile illnesses in Bolivia (*7*). The study was approved by the Naval Medical Research Unit No. 6 Institutional Review Board (Navy Medical Research Center Detachment 2000.0008) and conducted in collaboration with the Bolivia Ministry of Health.

Serologic analyses showed a 64-fold IgM seroconversion between the acute-phase (<100) and convalescent-phase samples (6,400) by using an IgM ELISA as described (*8*). Samples were also tested by ELISA for the following arboviruses: West Nile virus, dengue virus, Oropouche virus, Guaroa virus, Rocio virus, St. Louis encephalitis virus, yellow fever virus, Venezuelan equine encephalitis virus, and Mayaro virus. All test results were negative for these viruses. Virus isolation was attempted on the acute-phase serum sample by using Vero and C6/36 cells, but the culture did not yield any virus. Attempts to isolate virus by intracranial inoculation in suckling mice were also unsuccessful (University of Texas Medical Branch, Institutional Animal Care and Use Committee protocol 9505045).

Viral RNA was extracted from the acute-phase sample and reverse transcription PCR specific for a portion of the nonstructural protein 5 gene was performed by using a described method (*9*). A 189-bp PCR product was obtained, purified, and sequenced by using flavivirus primers FU1 and cFD2 (*9*) and further analyzed by using BLAST (www.ncbi.nlm.nih.gov/blast), resulting in ≈95% homology to ILHV. Phylogenetic analysis with neighbor-joining and parsimony methods grouped the nucleotide sequence of the ILHV virus from Bolivia with ILHV strains from Ecuador and Peru (Figure).

**Figure:**
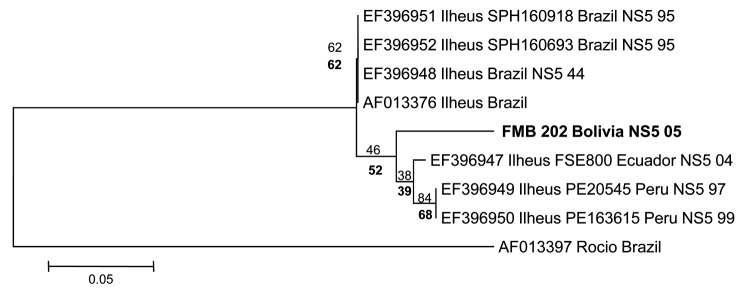
Phylogenetic analysis of the nonstructural protein 5 (NS5) gene region of 7 Ilheus virus isolates and a 189-bp nt sequence (FMB 202 Bolivia). Alignments were analyzed by using the neighbor-joining method with the Kimura 2-parameter algorithm in MEGA5 (www.megasoftware.net). Variation rate among sites was modeled with a gamma distribution (shape parameter = 1). Bootstrap confidence limits (from 1,000 replicates) are indicated at each node. Values in **boldface** below branches were obtained by parsimony analysis. **Boldface** indicates virus isolated in this study. Rocio virus (GenBank accession no. AF013397) was included as an outgroup on the basis of the phylogram of Kuno and Chang (*10*). Sequence generated in our study was deposited in GenBank under accession no. JN679229. Scale bar indicates nucleotide substitutions per site.

Magdalena is a tropical city in northern Bolivia that borders Brazil. The city is surrounded by rivers and chestnut fields, and agriculture and fishing are the main sources of employment. Despite having ecoepidemiologic conditions similar to those in other locations with a history of ILHV transmission, the virus had not been detected in the area. The patient had no travel history in the 30 days preceding his illness, indicating that the virus is probably endemic to the area.

Mild unspecific symptoms, a short viremic period, and lack of advanced confirmatory laboratory techniques in situ are some of the barriers impeding the diagnosis of ILHV in disease-endemic areas. High levels of antibody cross-reactivity among flaviviruses, which are also endemic to the area, might render diagnosis even more difficult. The presence of the main ILHV vector, *Psorophora* sp. mosquitoes, in the city suggests that much of the population that labors outdoors may be at risk for ILHV infection.

## References

[1] Laemmert HW, Hughes T. The virus of Ilheus encephalitis: isolation, serological specificity and transmission. J Immunol. 1947;55:61–7. 20285157

[2] Shope RE. Epidemiology of other arthropod-borne flaviviruses infecting humans. In: Chambers T, Monath T, editors. The flaviviruses: detection, diagnosis and vaccination development. Vol. 61. Amsterdam: Elsevier Academic; 2003. p. 386–7. 10.1016/s0065-3527(03)61009-2 14714437

[3] Nassar ES, Coimbra TL, Rocco IM, Pereira LE, Ferreira IB, de Souza LT, Human disease caused by an arbovirus closely related to Ilheus virus: report of five cases. Intervirology. 1997;40:247–52. 10.1159/000150554 9612726

[4] Spence L, Anderson CR, Downs WG. Isolation of Ilheus virus from human beings in Trinidad, West Indies. Trans R Soc Trop Med Hyg. 1962;56:504–9. 10.1016/0035-9203(62)90074-3 13990008

[5] Srihongse S, Johnson CM. Isolation of Ilheus virus from man in Panama. Am J Trop Med Hyg. 1967;16:516–8. 4378148 10.4269/ajtmh.1967.16.516

[6] Johnson BW, Cruz C, Felices V, Espinoza WR, Manock SR, Guevara C, Ilheus virus isolate from a human, Ecuador. Emerg Infect Dis. 2007;13:956–8. 17582910 10.3201/eid1306.070118 PMC2792834

[7] Forshey BM, Guevara C, Laguna-Torres VA, Cespedes M, Vargas J, Gianella A, Arboviral etiologies of acute febrile illnesses in Western South America, 2000–2007. PLoS Negl Trop Dis. 2010;4:e787. 10.1371/journal.pntd.0000787 20706628 PMC2919378

[8] Martin DA, Muth D, Brown T, Johnson A, Karabatsos N, Roehrig J. Standardization of immunoglobulin M capture enzyme-linked immunosorbent assays for routine diagnosis of arboviral infections. J Clin Microbiol. 2000;38:1823–6. 10790107 10.1128/jcm.38.5.1823-1826.2000 PMC86599

[9] Kuno G, Chang GJ, Tsuchiya KR, Karabatsos N, Cropp CB. Phylogeny of the genus *Flavivirus.* J Virol. 1998;72:73–83. 9420202 10.1128/jvi.72.1.73-83.1998 PMC109351

[10] Kuno G, Chang GJ. Biological transmission of arboviruses: reexamination of and new insights into components, mechanisms, and unique traits as well as their evolutionary trends. Clin Microbiol Rev. 2005;18:608–37. 10.1128/CMR.18.4.608-637.2005 16223950 PMC1265912

